# Cheiralgia Paresthetica or Superficial Radial Sensory Mononeuropathy: A Simple Diagnosis, A Simple Solution, and a Side Note on the Pathophysiology of the Tinel Sign

**DOI:** 10.7759/cureus.10224

**Published:** 2020-09-03

**Authors:** Hassan Kesserwani

**Affiliations:** 1 Neurology, Flowers Medical Group, Dothan, USA

**Keywords:** painful neuropathy, upper extremity trauma

## Abstract

We describe the case of a sculptor who developed superficial radial neuropathy (SRN) due to blunt trauma from striking a chisel for 30 years. The lesion was localized by the anatomical topography of the superficial radial nerve, a " hot " Tinel sign, and the graphic demonstration of reduced superficial radial sensory amplitude on a nerve conduction study (NCS). Our patient also responded to a strategically placed peripheral nerve block. We go further in this article and adumbrate on the underlying pathophysiology of the very Tinel sign we are so accustomed to, a clinical sign that is frequently deployed to diagnose a variety of peripheral nerve entrapments.

## Introduction

The superficial radial nerve is a pure sensory nerve, a branch of the radial nerve that arises from the bifurcation of the radial nerve in the proximal forearm as it leaves the arcade of Frohse and travels deep to the brachioradialis in the forearm. It emerges about nine centimeters (cm) proximal to the radial styloid, where it emerges between the brachioradialis and the extensor carpi radialis longus. Proximal to the wrist, it divides into two branches. The lateral branch lies one to three cm radial to Lister's tubercle (which is easily palpated on the dorsum of the radial styloid, around which the tendon of the extensor pollicis longus (EPL) acutely curves, akin to a pulley). The lateral branch supplies the radial side of the thumb. The medial branch passes directly over the EPL and supplies the first, second, and third web spaces in four digital divisions [1].

The nerve can be injured anywhere along its journey in the forearm. Blunt trauma from occupational exposure from a hammer or chisel, compression from the tight use of handcuffs, or even failed venipuncture of the cephalic vein of the forearm have been described [2]. It can also be seen as a manifestation of a mononeuritis multiplex [3]. Therapeutic modalities include the use of non-steroidal anti-inflammatory drugs, a short course of steroids, splinting of the wrist, local steroid injections in the vicinity of the " hot " Tinel sign at a focal point along the course of the nerve, and surgical decompression [4]. The Tinel sign is a frequently deployed clinical sign that helps with diagnosis and prognosis. Its definition, pathophysiology, and clinical relevance will be outlined in detail during the discussion. At this point, it suffices to mention that it has both diagnostic and prognostic value and is an essential part of the arsenal of clinical firepower at the fingertips of the clinician.

We describe a classic case of left superficial radial neuropathy (SRN) secondary to blunt trauma in an artisan who chronically chipped away at granite blocks. We graphically outline the lesion by localizing the Tinel sign and by demonstrating an amplitude drop of the superficial radial sensory nerve using a nerve conduction study. The patient responded to a strategically directed peripheral nerve block by deploying the Tinel sign for accurate localization of the site of injection. In this case report, we go further and discuss the pathophysiology of the Tinel sign and its value in clinical practice.

## Case presentation

We present the case of a 47-year-old right-handed sculptor, who has been chiseling away at granite blocks for cemetery headstones for the last 30 years. His artisanry involves clasping a block of granite with his bare left hand and chipping away at the block with a chisel in his right hand. On many occasions, the chisel inadvertently struck the region proximal to the radial styloid of his left distal forearm. One month prior to the presentation, he noted painful paresthesias when the volar and ulnar deviated his left wrist during the act of chiseling away at granite or when hyperpronating his forearm when swinging a club at a golf ball. He developed electric-like shock sensations over the skin of the radial styloid, dorsum of the thumb, and first web space of the left hand. He was able to localize a tender spot 3 centimeters (cm) exactly proximal to the Lister tubercle at the radial styloid of his left wrist. His occupation demanded active bimanual participation and he was eager for the rapid resolution of the problem. A short course of celecoxib 200 milligrams (mg) daily and a short course of ibuprofen 800 mg twice daily provided no relief.

His past medical history was significant for hypertension, hyperlipidemia, and diabetes. His medications included Atorvastatin, Januvia, Jardiance, and Lisinopril. 

**Figure 1:**
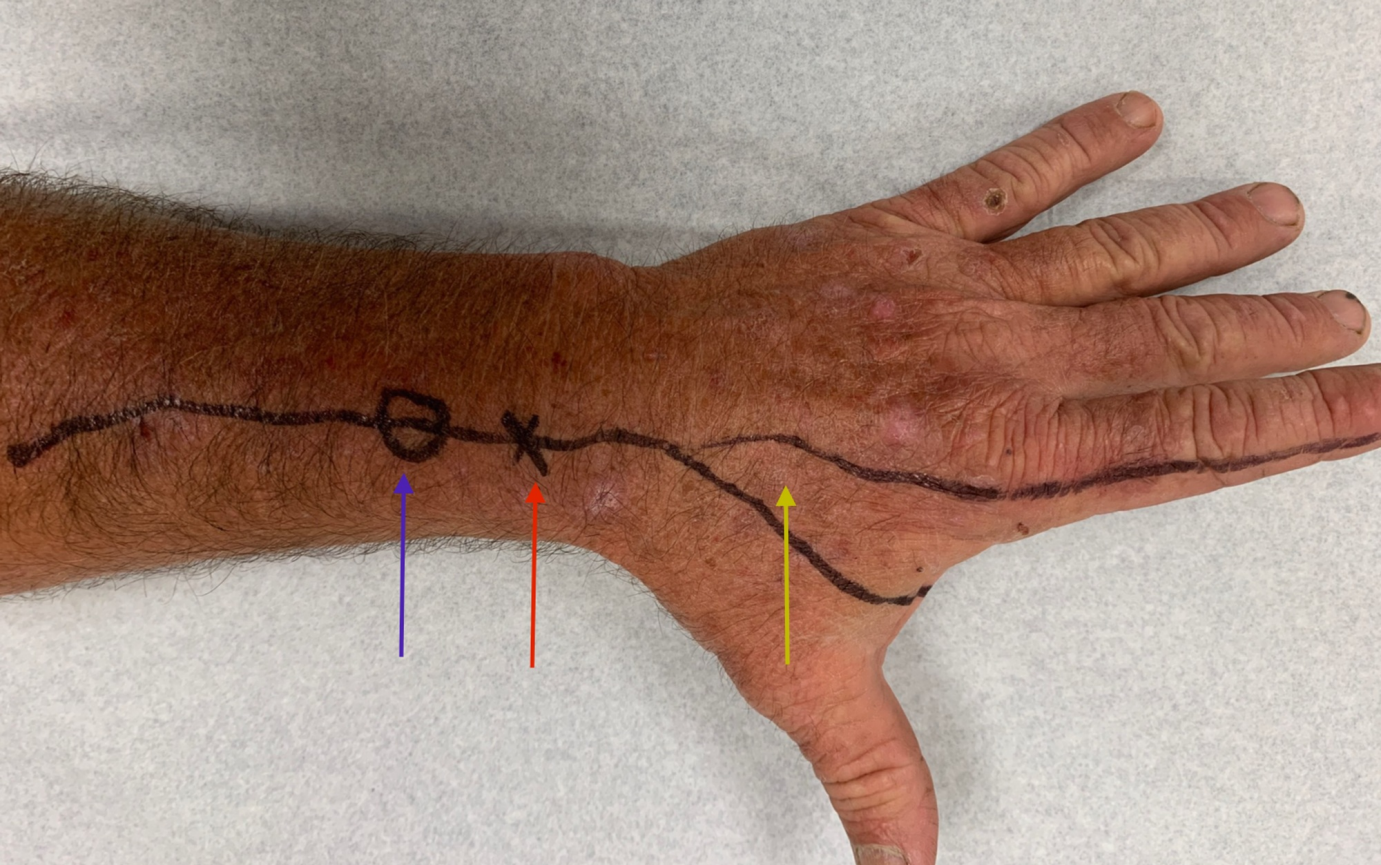
A photograph of the dorsum of the patient's left hand and distal forearm Locus of Tinel's sign (blue arrow), Lister's tubercle (red arrow), and area of dysesthesia over the first web space and radial aspect of the left index finger (yellow arrow). The distance between Lister's tubercle and the locus of Tinel's sign is 3 centimeters.

On examination, his height is 5 feet and 11 inches, with a weight of 196 pounds and a body mass index (BMI) of 27.3. His blood pressure was 110/78 with a pulse of 91. He appeared muscular and physically fit. His neurologic examination was normal except for dysesthesias to touch over the cutaneous distribution of the left superficial radial sensory nerve; over the left styloid, in a triangular region with apex 3 cm proximal to Lister's tubercle and over the first web space and radial aspect of the left index finger (Figure 1).

Percussion at a point 3 cm proximal to Lister's tubercle elicited a " hot " Tinel's sign with shooting electric pain into the thumb and index finger of the left hand. Pertinent normals include 5/5 motor strength by grading using the Medical Research Council (MRC) scale in both upper extremities. Specifically, deltoids, biceps, brachioradialis, triceps, extensor carpi radialis longus, extensor digitorum communis, extensor indices proprius, extensor pollicis longus, wrist flexors, finger spreaders, and finger flexors at the proximal and distal interphalangeal joints were entirely normal bilaterally. Deep tendon reflexes were symmetric and lively at the biceps, brachioradialis, and triceps bilaterally.

**Figure 2:**
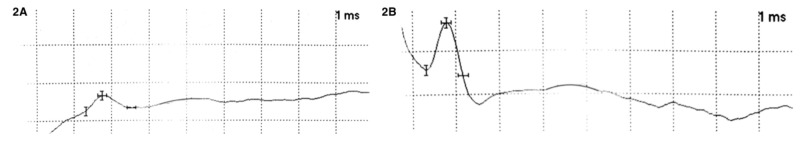
Self-evident drop in left superficial sensory amplitude 2A: Left superficial sensory amplitude measures 6.7 microVolts with a speed of 38.8 m/s; 2B: Right superficial sensory amplitude measures 24.3 microVolts with a speed of 45.5 m/s meters per second (m/s), millisecond (ms) NCS: Nerve Conduction Study: ordinate 20 microvolt per cell, abscissa 1 millisecond per cell

Clinically, a diagnosis of a left superficial radial sensory mononeuropathy was made. The next step necessitated a nerve conduction study (NCS) and electromyogram (EMG). Indeed, the NCS confirmed a definite drop in the left superficial radial sensory amplitude as compared to the right. This is demonstrated convincingly and graphically in Figure 2.

A superficial radial nerve block was performed using a 27 gauge needle. The needle is directed proximally along a line joining Lister's tubercle and the locus of Tinel's sign. Three milliliters (ml) of 1% xylocaine is injected subcutaneously in the vicinity of Tinel's locus. Special care is taken to avoid the nerve and the cephalic vein. Immediate numbness should be obtained over the cutaneous distribution of the nerve. Indeed, the patient responded to the peripheral nerve block and was pain-free at a follow-up visit one month later.

## Discussion

The Tinel sign is the development of paresthesia evoked in the distal sensory distribution of a damaged peripheral nerve and is elicited by percussion of the nerve more proximally. It indicates the compression (with or without demyelination) or regeneration of peripheral nerve fibers (axonal sprouting). It is useful as a diagnostic tool, as in percussion of the median nerve across the carpal tunnel in carpal tunnel syndrome or across the peroneal motor nerve in the fibular tunnel in common peroneal nerve entrapment at the fibular tunnel. The Tinel sign is useful for tracing the path of recovery or peripheral nerve regeneration along the course of a nerve and across the site of injury from proximal to distal [4].

A few definitions will clarify the next paragraph. By polymodal nociceptors, we mean nociceptors that respond to mechanical, thermal, and chemical stimuli in the noxious range. By mechanoreceptors, we mean receptors that respond to mechanical pressure or distortion and this includes Pacinian corpuscles (vibration and pressure), Meissner's corpuscle (light touch and low-frequency vibration 10-50 Hertz), Merkel nerve endings (pressure, position and deep static touch, shapes, and edges), and Ruffini corpuscles (skin stretch and kinesthetic sense). By thermoreceptors we mean receptors that code for an absolute or relative change in temperature in the innocuous range. These include the slow unmyelinated C-fibers that respond to warmth and cold and the faster A delta fibers that respond to cold.

A crush lesion of a murine sural nerve is followed by sprouting of large myelinated A-fibers and small unmyelinated C-fibers into the distal nerve stump. Almost all of the myelinated A-fibers remain mechanosensitive and 56% of the C-fibers remain thermosensitive to cold and heat [5]. Histologically, axotomy of peripheral nerves is followed by sprouting of axons across the site of injury and along the Schwann cell basement membrane tubes. This recovery involves both mechanical (touch, pressure, vibration) and thermal sensations (cold, heat). However, functional recovery is better after a crush lesion than after nerve transection and suture because axons regenerate along their original endoneurial Schwann cell tubes, which are usually interrupted by a transection. After transection and suture of a peripheral nerve, 30% of the unmyelinated axons die, leading to a decrease in the number of axons distal to the lesion. Regeneration of unmyelinated axons has shown that low-threshold mechanoreceptors and cold thermoreceptors have normal properties, whereas polymodal nociceptors exhibit changes of threshold. Between four and 21 days following a sural nerve lesion (crush, transection, and re-suturing), many regenerating axons exhibit ectopic activity and responses to mechanical and thermal stimuli applied to the peripheral nerve along the path of regeneration. Myelinated A-fibers are predominantly mechanosensitive; some are spontaneously active and a few could be excited by heat stimuli applied to the lesioned peripheral nerve. However, 60% of the regenerating unmyelinated C-fibers demonstrate active ectopic activity or mechanoreceptor and heat/cold sensitivity. The remaining 40% of the unmyelinated C-fibers have some ectopic activity. These persistent ectopic and evoked discharges in lesioned nerve fibers are important in the generation of spontaneous evoked dysesthesias and paresthesias and may explain the Tinel sign with percussion (mechanoreception) [6]. In summary, myelinated A-fibers recovery is complete following peripheral nerve injury. But unmyelinated C-fibers show ectopic activity and abnormal function.

Running superficially near a flexible joint, the superficial radial sensory nerve is prone to injury. Blunt trauma and damage from venipuncture of the cephalic vein are two such examples [2]. It can also be involved as part of a mononeuritis multiplex as seen with Wartenberg's migrant sensory neuropathy [3]. A neuroma can form after trauma and, rarely, a schwannoma can be an etiology [7]. Sarcoidosis manifesting as a mononeuritis multiplex with the involvement of the superficial radial sensory can be etiologic too (personal observation). In a study of 51 patients with entrapment of the superficial radial sensory nerve, 19 patients were treated conservatively of which 37% improved. Thirty-two patients were treated with surgical decompression, of which 86% improved [8]. Once conservative measures, such as splinting, non-steroidal anti-inflammatory agents, or a short course of steroids, fail, a peripheral nerve block near the focal point of the Tinel's sign is recommended. Three cc of a local anesthetic, xylocaine 1%, is injected subcutaneously with a 27 gauge needle directed proximally along the course of the nerve, with special care taken to avoid a direct hit on the superficial radial sensory nerve or cephalic vein. This is easy to avoid, as directly hitting the nerve elicits a sharp electric pain, which necessitates needle withdrawal. Should a peripheral nerve block fail, the clinician resorts to surgical decompression of the nerve. In some instances, transection of the nerve as it exits the overlapping brachioradialis and the extensor carpi radialis longus may be necessary [9].

## Conclusions

The diagnosis of SRN is a simple exercise. Its etiology and treatment algorithm are straightforward. When isolated, its treatment is well-defined. When due to an underlying condition, such as a Wartenberg's migrating neuritis or other mononeuritis multiplex, one treats the underlying condition. Despite being a simple clinical sign, the pathophysiology of the Tinel sign in peripheral nerve injury is insightful for understanding the underlying mechanisms and recovery dynamics of peripheral nerve injuries. In particular, when a nerve is crushed, the myelinated A-fibers regenerate through the Schwann cell tube but the unmyelinated C-fibers recover incompletely and the axonal sprouts develop ectopic activity, which explains the Tinel sign. One can also follow the regenerating peripheral nerve by tracing the displacement of the focal point of the Tinel sign over time, distal to the site of injury. This case also points out the usefulness of comparing the sensory nerve action potential on both sides in order to improve the accuracy of electrophysiological diagnostic testing.
